# Phase I dose escalation and expansion trial of single agent ONC201 in pediatric diffuse midline gliomas following radiotherapy

**DOI:** 10.1093/noajnl/vdac143

**Published:** 2022-09-13

**Authors:** Sharon L Gardner, Rohinton S Tarapore, Jeffrey Allen, Susan L McGovern, Wafik Zaky, Yazmin Odia, Doured Daghistani, Zuanel Diaz, Matthew D Hall, Ziad Khatib, Carl Koschmann, Evan Cantor, Ryo Kurokawa, Tobey J MacDonald, Dolly Aguilera, Nicholas A Vitanza, Sabine Mueller, Cassie Kline, Guangrong Lu, Joshua E Allen, Soumen Khatua

**Affiliations:** NYU Langone Medical Center and School of Medicine, New York, NY, USA; Department of Pediatrics, NYU Grossman School of Medicine, New York, NY, USA; The University of Texas MD Anderson Cancer Center, Houston, TX, USA; Clinical Development, Oncoceutics, Philadelphia, PA, USA; Clinical Development, Chimerix Inc., Durham, NC, USA; NYU Langone Medical Center and School of Medicine, New York, NY, USA; The University of Texas MD Anderson Cancer Center, Houston, TX, USA; The University of Texas MD Anderson Cancer Center, Houston, TX, USA; Miami Cancer Institute, Baptist Health South Florida, Miami, FL, USA; Miami Cancer Institute, Baptist Health South Florida, Miami, FL, USA; Miami Cancer Institute, Baptist Health South Florida, Miami, FL, USA; Miami Cancer Institute, Baptist Health South Florida, Miami, FL, USA; Department of Radiation Oncology, Nicklaus Children’s Hospital, Miami, FL, USA; Department of Radiation Oncology, Miami Cancer Institute, Baptist Health South Florida, Miami, FL, USA; Department of Neuro-Oncology, Nicklaus Children’s Hospital, Miami, FL; Michigan Medicine, University of Michigan Medical School, Ann Arbor, MI, USA; Michigan Medicine, University of Michigan Medical School, Ann Arbor, MI, USA; University of Connecticut School of Medicine, Farmington, CT; Michigan Medicine, University of Michigan Medical School, Ann Arbor, MI, USA; Children’s Healthcare of Atlanta, Emory University School of Medicine, Atlanta, GA, USA; Children’s Healthcare of Atlanta, Emory University School of Medicine, Atlanta, GA, USA; Ben Towne Center for Childhood Cancer Research, Seattle Children’s Research Institute, Seattle, WA, USA; Department of Pediatrics, Seattle Children’s Hospital, University of Washington, Seattle, WA, USA; Department of Neurology, Neurosurgery and Pediatrics, University of California San Francisco; Department of Pediatrics, University of California San Francisco, San Francisco, CA, USA; Department of Neurology, University of California San Francisco, San Francisco, CA, USA; Clinical Development, Oncoceutics, Philadelphia, PA, USA; Clinical Development, Oncoceutics, Philadelphia, PA, USA; Clinical Development, Chimerix Inc., Durham, NC, USA; Pediatric Hematology/Oncology, Mayo Clinic, Rochester, MN, USA

**Keywords:** diffuse midline glioma, H3 K27M-mutant glioma (DMG), diffuse intrinsic pontine glioma (DIPG), ONC201

## Abstract

**Background:**

ONC201, a dopamine receptor D2 (DRD2) antagonist and caseinolytic protease P (ClpP) agonist, has induced durable tumor regressions in adults with recurrent H3 K27M-mutant glioma. We report results from the first phase I pediatric clinical trial of ONC201.

**Methods:**

This open-label, multi-center clinical trial (NCT03416530) of ONC201 for pediatric H3 K27M-mutant diffuse midline glioma (DMG) or diffuse intrinsic pontine glioma (DIPG) employed a dose-escalation and dose-expansion design. The primary endpoint was the recommended phase II dose (RP2D). A standard 3 + 3 dose escalation design was implemented. The target dose was the previously established adult RP2D (625 mg), scaled by body weight. Twenty-two pediatric patients with DMG/DIPG were treated following radiation; prior lines of systemic therapy in addition to radiation were permitted providing sufficient time had elapsed prior to study treatment.

**Results:**

The RP2D of orally administered ONC201 in this pediatric population was determined to be the adult RP2D (625 mg), scaled by body weight; no dose-limiting toxicities (DLT) occurred. The most frequent treatment-emergent Grade 1-2 AEs were headache, nausea, vomiting, dizziness and increase in alanine aminotransferase. Pharmacokinetics were determined following the first dose: *T*_1/2_, 8.4 h; *T*_max_, 2.1 h; *C*_max_, 2.3 µg/mL; AUC_0-tlast_, 16.4 hµg/mL. Median duration of treatment was 20.6 weeks (range 5.1-129). Five (22.7%) patients, all of whom initiated ONC201 following radiation and prior to recurrence, were alive at 2 years from diagnosis.

**Conclusions:**

The adult 625 mg weekly RP2D of ONC201 scaled by body weight was well tolerated. Further investigation of ONC201 for DMG/DIPG is warranted.

**Key Points**

- H3 K27M-mutant glioma is fatal and has no effective treatments beyond radiation.
- ONC201, a novel imipridone, has shown efficacy in adult H3 K27M-mutant glioma
- In pediatric H3 K27M-mutant glioma, ONC201 was safe and well-tolerated

**Importance of the Study**

Durable objective responses in adults with recurrent H3 K27M-mutant DMG were reported with single agent ONC201, a small molecule DRD2 (dopamine receptor D2) antagonist and ClpP (caseinolytic mitochondrial matrix peptidase proteolytic subunit) agonist. Given that DMGs often occur in children and there are no effective therapies following first-line radiation, the first pediatric clinical trial of ONC201 was conducted. The study confirmed the adult recommended phase II dose administered weekly by oral capsules to be well tolerated in pediatric patients when the dose is scaled by body weight. Results for this study indicate a similar safety and pharmacokinetic profile of ONC201 to those observed in adults.

Diffuse intrinsic pontine glioma (DIPG) is predominantly a pediatric brain tumor with a median age of 6 years at diagnosis and an overall survival of 11 months.^1^ While numerous clinical trials have been conducted with treatment interventions spanning radiation, chemotherapy, and targeted agents, little improvement in clinical outcomes has been achieved.

In 2016, the World Health Organization classified H3 K27M-mutant diffuse midline glioma (DMG) as a distinct form of Grade IV glioma, regardless of histological features, which is associated with an extremely poor prognosis.^2^ In an analysis of tumor samples from pediatric patients with glial and glioneuronal tumors, the proportion of patients harboring a H3 K27M mutation was 91% in DIPG and 45% in thalamic glioma.^3^ Standard therapy for DMG/DIPG involves external beam radiotherapy with doses of 50-54 Gy fractionated over 6 weeks.^4^ Gross total resection of DMG/DIPG is not feasible due to location.^4^ Single-agent temozolomide, which is indicated for adult anaplastic astrocytoma and glioblastoma,^5^ has not demonstrated improved efficacy in the treatment of DIPG^6^ and its utility in H3 K27M-mutated DMG is likely limited given that these tumors are predominantly MGMT unmethylated.^7^

Dopamine receptor D2 (DRD2) is a G protein-coupled receptor that can promote tumor growth in a broad range of malignancies and has emerged as a therapeutic target for gliomas and other tumors that overexpress this receptor.^8,9^ ONC201 is a small molecule bitopic antagonist of DRD2/3^10,11^ and an allosteric agonist of the mitochondrial protease caseinolytic mitochondrial matrix peptidase proteolytic subunit (ClpP).^12^ The compound crosses the blood–brain barrier and exhibits p53-independent anti-cancer efficacy in preclinical models of high-grade glioma.^13–15^ Downstream of target engagement, ONC201 also activates the ATF4/CHOP-mediated integrated stress response pathway,^16–18^ inactivates Akt/ERK signaling,^14^ and degrades c-myc.^16^ These effects often induce DR5/TRAIL-mediated apoptosis of tumor cells; however, the phenotype can vary by tumor cell type to also include DR5/TRAIL-independent apoptosis, cell cycle arrest, or general antiproliferative effects.^14,18,19^

Results from the first phase II clinical trial of oral ONC201 in adults with recurrent, bevacizumab-naïve glioblastoma were previously reported using the recommended phase II dose of 625 mg administered orally once every 3 weeks (NCT02525692).^20^ Among this initial cohort of 17 patients, a 22-year-old woman with a thalamic glioma that harbored a biopsy-proven H3 K27M mutation experienced a near-complete durable response, including complete regression of the primary thalamic lesion. Preclinical studies demonstrated that glioma cell lines harboring the H3 K27M mutation were more susceptible to ONC201 cytotoxicity than wild-type counterparts, and that this increased sensitivity was coincident with elevated DRD2 expression.^21^ Based on these clinical and preclinical findings, a series of clinical investigations were initiated to evaluate ONC201 in patients with H3 K27M-mutated disease. Two phase II clinical trials in adults with recurrent DMG treated with ONC201 monotherapy were initiated (NCT03295396; NCT02525692), as well as the first phase I pediatric clinical trial of ONC201. The objective of the present pediatric trial was to determine the safety and recommended phase II dose (RP2D) of ONC201 as a single agent in pediatric patients with DMG following standard of care radiation (NCT03416530).

## Materials and Methods

### Patient Population and Protocol Design

The primary objective of this open label study was to determine the RP2D of single-agent ONC201 in pediatric patients with glioma. The secondary objectives included safety and tolerability, efficacy, and the pharmacokinetic (PK) profile of ONC201 in pediatric patients.

Eligible patients were 2 to <19 years of age, diagnosed with H3 K27M-mutant glioma who had completed prior radiotherapy, were at least 4 weeks from prior cytotoxic therapy, and had a minimum body weight of 10 kg (complete eligibility criteria provided in Supplementary Table S1). Patients were not required to have recurrent disease at baseline given the primary objective of safety. Evidence of the H3 K27M mutation by immunohistochemistry or sequencing must have been assessed in a Clinical Laboratory Improvement Amendment-certified laboratory or equivalent setting. Two DIPG patients were permitted to enroll without biopsy given the high prevalence of H3 K27M in this location, guardian concern over biopsy, and consent to biopsy at autopsy. Patients were required to have adequate organ function and a baseline Karnofsky performance status (KPS) or Lansky performance status (LPS) > 50. The time of first and last provision of signed consent was January 25, 2018 and September 4, 2018, respectively.

ONC201 capsules were orally administered once a week. The target adult dose of 625 mg was allometrically scaled as shown in Supplementary Table S2; briefly, dose for each weight was calculated using a power model assuming an average adult weight of 70 kg and an exponent of 3/4.^22^ Doses were rounded to the nearest 125 mg (strength of one capsule). The specific dose of ONC201 was dependent on the body weight of the patient and the designated dose level. The body weight was rounded to the nearest 5 kg interval with consideration of adjusted weight for patients on steroids. If body weight changed during the clinical trial, the number of ONC201 capsules per dose was reassessed at the beginning of a given treatment cycle (21-day period). RP2D was defined as the maximum tolerated dose or maximum administered dose (MAD) that is targeted to achieve the equivalent of the adult 625 mg dose (dose level 2) as the MAD. Dose limiting toxicity (DLT) was defined as any ≥ Grade 3 drug-related adverse event (AE) or abnormal lab value that occurred in the first cycle (i.e. 21 days) of treatment. The definition of DLT required the AE to be unrelated to disease, disease progression, intercurrent illness, or concomitant medications, and judged by the investigator to be “possibly related”, “probably related”, or “definitely related” to ONC201.

Treatment beyond disease progression was permitted if it was considered in the best interest of the patient. This treatment was permitted to include bevacizumab and/or radiotherapy.

Dose escalation began at one 125 mg capsule less than the adult RP2D scaled by body weight according to the standard 3 + 3 design without incidence of DLT. After completion of the 21-day DLT window by six patients at the adult RP2D scaled by body weight, an additional 13 patients were enrolled in the RP2D dose expansion cohort to expand the safety and pharmacokinetic experience at the RP2D.

Plasma (approximately 1 mL) was collected in all patients at multiple timepoints during cycle 1 (pre-dose and 0.5, 2, 4, 24, 48, 168, 336 hours after first ONC201 administration) and prior to dosing on Day 1 on even-numbered subsequent cycles to assess PK. ONC201 plasma concentrations were analyzed using 50 mL of K_2_EDTA plasma by a validated LC/MS-MS method^23^ with positive ESI-MRM mode that has a linear range of 1 to 500 ng/mL (samples were diluted if the limit of detection). Deuterated ONC201 was used as an internal standard.

## Study Evaluations

The data cutoff date was October 15, 2020. History and physical examinations, complete blood count, comprehensive metabolic panel, magnesium, phosphorus, and urinalysis were performed every three weeks. All adverse events were assessed using NCI Common Terminology Criteria for Adverse Events (CTCAE) version 5 and were recorded from the time the date of initiation of study therapy through 30 days following cessation of treatment. At baseline, a standard 12-lead electrocardiogram (ECG) was performed for all enrolled patients. On-treatment ECGs were performed on patients if deemed necessary by the investigator. Gadolinium-enhanced MRI was performed at baseline and subsequently every eight weeks. The MRI protocol included axial and sagittal T1-weighted images with coronal FLAIR, axial dual echo, coronal inversion recovery T2 and axial, sagittal and coronal T1 post-gadolinium sequences. Tumor assessments were conducted by investigator. Response Assessment in Neuro-Oncology (RANO) criteria were used for tumor evaluations in patients with enhancing tumors. For patients with non-enhancing tumors, tumor evaluations were performed using RANO-low grade glioma criteria. Progression-free survival (PFS) was defined as time from first dose of ONC201 to radiologic or clinical progression or death. Among patients treated with ONC201 beyond progression, imaging continued at the regular intervals; among those continuing with the addition of radiation, imaging resumed at least eight weeks after completion of radiotherapy.

ONC201 PK parameters in plasma and tumor were calculated using noncompartmental analysis. Continuous safety and PK (plasma and tumor biopsy) data were summarized descriptively. Discrete PK parameters (e.g. *T*_max_) were presented as median and range (minimum, maximum).

### Ethics Approval and Consent to Participate

The protocol and informed consent form were approved by central institutional review board (IRB) and as required by individual sites, local IRBs. Patients provided written informed consent with assent obtained when appropriate based on the patient’s age. The study was conducted in accordance with the ethical principles originating in the Declaration of Helsinki and was consistent with International Conference on Harmonization/Good Clinical Practice guidelines and applicable regulatory requirements. The study is registered on clincialtrials.gov: NCT03416530.

## Results

### Patient characteristics

Demographic and clinical characteristics are provided in Table 1. Twenty-two patients were enrolled with a median age of 8 (range 2-18) years old. Sixteen patients (72.7%) had disease that was not recurrent at enrollment while the remaining 6 patients (27.3%) had recurrent disease. Eleven had DIPG with H3 K27M-mutation, 2 had DIPG with unknown H3K27M-mutation status, and 9 had H3 K27M-mutant DMGs located outside of the pons.

**Table 1.** Demographics and disease characteristics of patient population

	Allpatients (*N* = 22)
Gender, *n* (%)	
Female	12 (55)
Male	10 (45)
Age, years, median (range)	8 (2-18)
Weight, kg, median (range)	38.8 (11.8-135.9)
LPS/KPS, median (range)	80 (50-90)
Primary tumor location, *n*(%)	
Pons	13 (59.1)
Thalamus	5 (22.7)
Cerebellum	3 (13.6)
Brainstem (non-pontine)	1 (4.5)
Diagnosis, *n* (%)	
DMG, H3 K27M-mutant	20 (91)
DIPG (biopsied)	11 (50)
Non-DIPG (biopsied)	9 (41)
DIPG (non-biopsied)	2 (9)
Multifocal disease, *n* (%)	
Yes	4 (18)
No	18 (82)
Disease status, *n* (%)	
Not recurrent	16 (72.7)
Recurrent	6 (27.3)
H3 K27M mutation, *n* (%)	
IHC	10 (45.4)
NGS	10 (45.4)
H3.3 K27M-mutant	8 (36.4)
H3.1 K27M-mutant	2 (9.1)
Unknown	2 (9.1)
Number of lesions, median, (range)	1 (1-2)
Prior lines of therapy, median (range)	1.5 (1-3)
Time from diagnosis, weeks, median, (range)	22.9 (11-147)
Time from prior radiation, weeks, median, (range)	11.6 (1-79.7)
Re-irradiation, *n* (%)	1 (4.5)
Dexamethasone, mg/day, median (range)	2 (0.25-12)
Ethnicity	
White	14 (63.6)
Unknown	5 (22.7)
Other	1 (4.5)
Asian	1 (4.5)
Black or African American	1 (4.5)
Concomitant medication, *n*(%)	
Cannabidiol oil	2 (9.1)

DIPG, diffuse intrinsic pontine glioma; DMG, diffuse midline glioma; IHC, immunohistochemistry; KPS, Karnofsky performance status; Lanksy performance status; NGS, next generation sequencing.

All patients received at least one prior line of therapy (median 1.5; range 1-3), all of which included radiation. Median time from diagnosis was 22.9 weeks (range 11-147) and median time from completion of prior radiation was 11.6 weeks (range 1-79.7). One patient underwent re-irradiation before starting on ONC201.

All patients received at least three doses of ONC201 and thus completed the DLT window, except one on account of rapid disease progression. As of October 15, 2020, patients received a median of 18 once-weekly doses of therapy (range 3-41).

### Safety

Table 2 lists the observed treatment-emergent AEs (TEAE). No DLTs or treatment discontinuation due to ONC201-related toxicity occurred in any patient. The most frequent Grades 1 and 2 TEAEs were nausea (22.7%), vomiting (18.2%), headache (13.6%), and increase in alanine aminotransferase (13.6%; Table 2). The most frequent grade 3/4 TEAEs were neutropenia (13.6%), increased aspartate aminotransferase (4.5%), and decreased lymphocyte count and decreased white blood cell count (4.5%; Table 2).

**Table 2.** Treatment-emergent adverse events irrespective of attribution (>10% patients)

System organ class, *n* (%) Preferred term	All grades (*N*%)	Grade 3 or 4 *N* (%)
Patients with at least one TEAE	21 (95.5)	12 (54.4)
Nervous system disorders	20 (90.9)	3 (13.6)
Headache	12 (54.5)	0
Hemiparesis	4 (18.2)	1 (4.5)
Dysarthria	3 (13.6)	0
Ataxia	3 (13.6)	0
Dizziness	6 (27.3)	0
Gastrointestinal disorders	12 (54.5)	1 (4.5)
Vomiting	6 (27.3)	0
Nausea	8 (36.4)	0
Abdominal pain	4 (18.2)	1 (4.5)
Dyspepsia	3 (13.6)	0
General disorders and administration sitecondition	10 (45.5)	1 (4.5)
Fatigue	8 (36.4)	0
Musculoskeletal and connective tissue disorders	8 (36.4)	0
Back pain	3 (13.6)	0
Muscular weakness	3 (13.6)	0
Investigations	6 (27.3)	2 (9.1)
Alanine aminotransferase increased	3 (13.6)	0
Eye disorders	5 (22.7)	0
Diplopia	3 (13.6)	0
Respiratory, thoracic and mediastinal disorders	6 (27.3)	2 (9.1)
Cough	3 (13.6)	0

TEAE, treatment-emergent adverse event.

Three patients had Grade 3 neutropenia that was attributed as possibly or probably related to ONC201. Two patients, a 3-year-old male (dose level 2) and a 5-year-old male (dose level 1), had dose withheld for 1-2 weeks until the possibly related neutropenia was resolved. Another patient, an 8-year-old male (dose level 2), had a dose reduction by one dose level on account of probably related neutropenia. The neutropenic event occurred 45 weeks after beginning ONC201, resolved within a 7-day period, and the patient continued on-treatment for another 34 weeks until progression.

Concomitant administration of cannabidiol (CBD) oil while on ONC201 was documented in two patients. While co-administering CBD oil with ONC201, one patient experienced transient probably related Grade 1 vomiting.

A 5-year-old female patient had dizziness and ataxia approximately 20 min after drug administration that lasted for approximately 45 min and occurred only at the time of drug administration. This was observed at the time of first drug administration and recurred with subsequent doses. Due to ataxia and dizziness, the patient was on bedrest for about an hour after subsequent doses. The patient had a *C*_max_ of 6.5 µg/mL (approximately 3-fold higher than mean *C*_max_) at 30 minutes after drug administration.

### Pharmacokinetics

**Figure 1.**
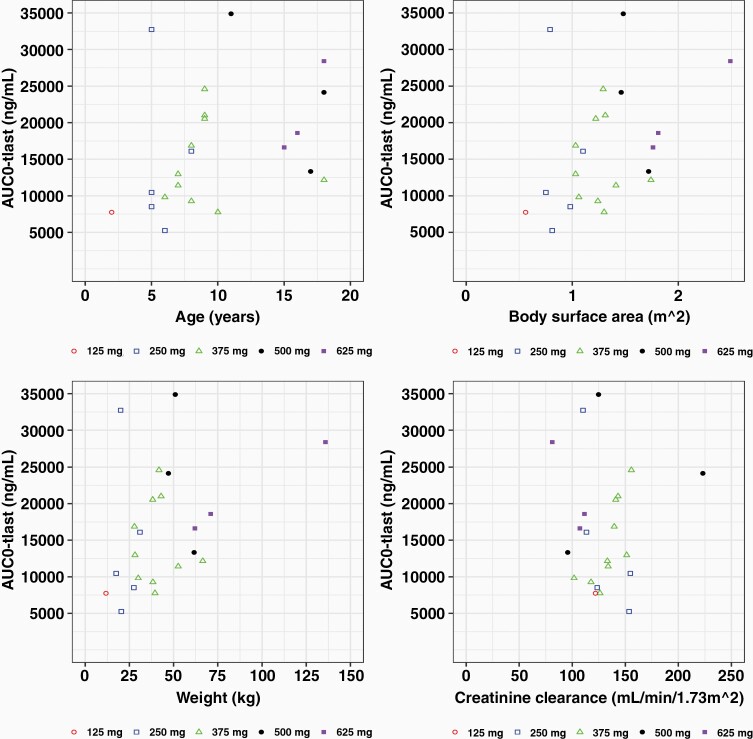
Pharmacokinetic analysis of weekly, oral ONC201 in pediatric H3 K27M-mutant glioma patients. ONC201 exposure covariance with AUC. Each dot represents AUC for each patient.

Pharmacokinetics were assessed in plasma samples collected at serial time points during the first 3 weeks of ONC201 administration (Table 3; Supplementary Fig. 1). A trend of dose-proportionate exposure was observed with a mean half-life of 8.4 h and a *C*_max_ of 2.3 µg/mL (~5.9 µM; SD, 1.3 µg/mL), which occurred at 2.1 h following administration (*T*_max_). *C*_max_ exceeded the preclinical therapeutic threshold of 1 µg/mL based on DRD2/ClpP target engagement and anti-cancer activity.^10–12,14^ The mean volume of distribution was 187 L (SD, 138L), consistent with a large distributive volume. Mean AUC_0-tlast_ was 16.4 h. µg/mL (SD, 8.3 h. µg/mL) and mean CL was 27.5 L/h (SD 12.7 L/h). Generally, CL/F was observed to be variable among patients, but generally consistent across dose groups (Fig. 1 and Supplementary Fig. S2). There were no apparent relationships between drug CL/F and patient sex and age. Exposure (AUC and *C*_max_) varied by age, body weight, and body surface area (Fig. 1 and Supplemental Fig. S2).

**Table 3.** Pharmacokinetic parameters of ONC201 after the first dose (*N* = 22)

Parameter	Mean	SD	Median	Minimum	Maximum
*C*_max_ (µg/mL)	2.3	1.3	1.9	0.9	6.8
*T*_max_ (h)	2.1	1.25	2	0.5	4
AUC_0-tlast_ (µgh/mL)	16.4	8.3	14.7	5.3	34.9
AUC_0-inf_ (µgh/mL)	16.5	9.4	13.6	5.3	35.8
T_1/2_ (h)	8.37	6.19	6.26	2.89	24.5
CL (L/h)	27.5	12.7	23.9	7.58	52.8
Vd (L)	187	138	140	64.4	546

AUC_0-inf_, area under the curve from time 0 to infinity; AUC_0-tlast_, area under curve from time 0 to *last* time point with measurable concentration; CL, clearance; *C*_max_, maximum serum concentration; SD, standard deviation; T1/2, half-life; *T*_max_, time to *C*_max_; Vd, volumne of distribution.

### Clinical Outcomes

**Figure 2.**
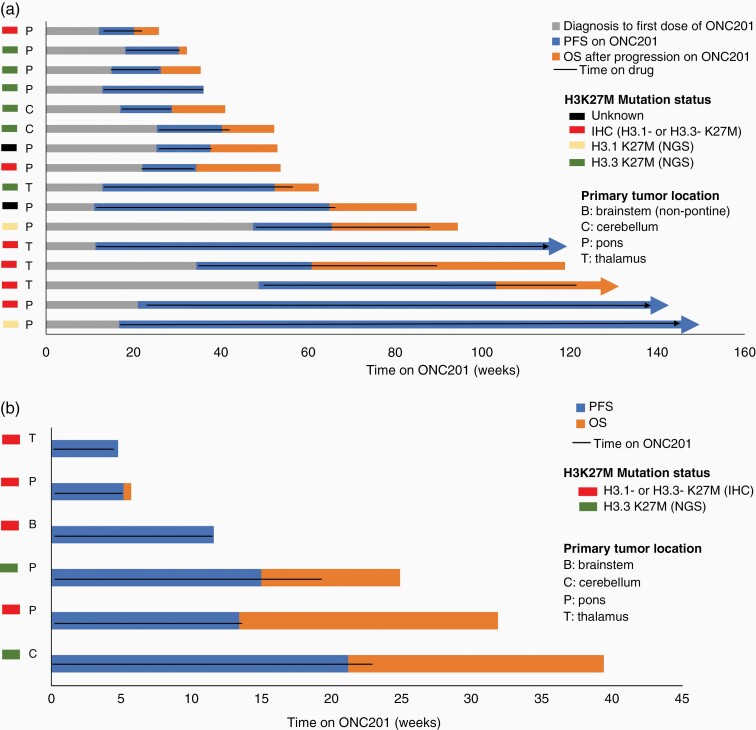
Clinical outcomes of ONC201-treated pediatric H3 K27M-mutant glioma patients. Swimmer plot of patients who (**a**) initiated ONC201 following radiation but prior to recurrence or (**b**) after recurrence. The black line indicates time on ONC201.

For patients who initiated ONC201 following radiation and prior to recurrence, median PFS was 20.4 weeks (range, 7.8-129; Supplementary Table S3) from starting ONC201; and 3 patients remain on therapy (Fig. 2a). Median OS was 53.8 weeks (range, 25.6-145.5; Supplementary Table S3) from diagnosis and overall survival at 24 months was 22.7%. Median time from diagnosis to ONC201 initiation was 22.9 weeks (range 11.2-48.7).

**Figure 3.**
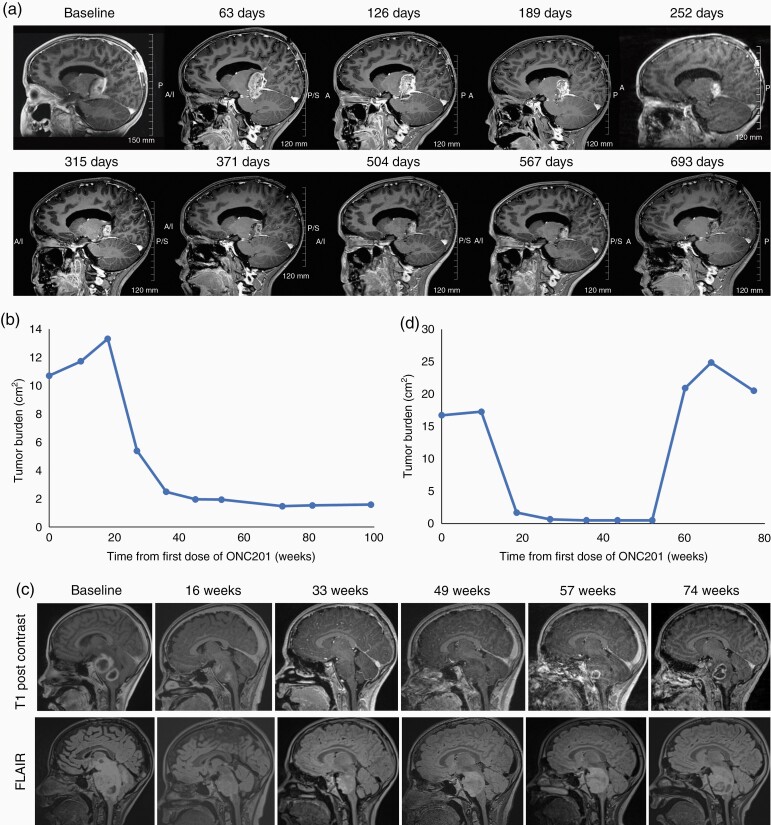
Radiographic evaluation of ONC201 in pediatric patients with H3 K27M-mutant thalamic glioma (a, b) or DIPG (c, d) who had previously received radiation. (**a**) Sagittal T1 post-contrast MRI images and (**b**) tumor size in enrolled patient prior to and following ONC201 treatment. Baseline MRI is 2 days after completion of radiation and ONC201 treatment began 7 days after completion of radiation. (**c**) Sagittal T1 post-contrast and T2/FLAIR MRI images of the primary tumor at baseline and at 19, 36, 52, and 60 weeks after beginning ONC201 treatment of a 9-year-old male with DIPG who began ONC201 4.5 weeks after completing radiation therapy and (**d**) radiographic change in tumor burden from baseline.

A 6-year-old female patient with an H3 K27M-mutant thalamic glioma began ONC201 one week after completing radiation therapy. The patient experienced an 87% regression in tumor size (Fig. 3a, b) that has remained durable and she has continued on therapy for  > 2 years. Another patient, a 9-year-old male with H3 K27M-mutant DIPG, began ONC201 therapy 4.5 weeks after completing radiation therapy. The patient achieved a radiographic response 18.5 weeks after initiating ONC201 that was durable for an additional 41.7 weeks until progression (Fig. 3c, d). The patient was then treated beyond progression for an additional 17.8 weeks. Another patient, an 8-year-old female with H3 K27M-mutant DIPG began ONC201 therapy 8 weeks after completing radiation therapy. The patient has achieved a 72% reduction in tumor size after 88 weeks on ONC201 and continues on therapy. The patient also had clinical improvement outside of the expected window for radiation therapy benefit, as documented by her baseline LPS improving to 100 after 54 weeks of ONC201 from 80 at baseline.

The six patients with prior radiation and recurrent disease at ONC201 initiation (Fig. 2b) had a median PFS of 12.6 weeks (range, 4.8-21.3) and overall survival of 19.6 weeks (range 4.8-39.5) relative to initiation of ONC201 (Supplementary Table S3).

## Discussion

DIPGs and DMGs are invariably lethal with no proven curative therapies. First-line radiation is thought to extend overall survival by 3-6 months, though progression is inevitable. The identification of the H3 K27M mutation in the vast majority of DIPG and pathognomonic for DMGs has created an avenue for the approach of targeted therapies in this population, however clinically effective therapies remain elusive.

DMGs have been reported to harbor elevated expression of DRD2,^9^ potentially as a downstream epigenetic consequence of the mutation. ONC201 is well suited to address this potential vulnerability as a selective DRD2 antagonist that exhibits blood-brain-barrier penetrance^24^ and p53-independent anti-cancer efficacy.^14^ Interestingly, DRD2 expression within the central nervous system is highest in midline structures of the brain (https://www.proteinatlas.org/ENSG00000149295-DRD2/brain),^25^ where the H3 K27M mutation is present and bystander effects of ONC201 have been documented in preclinical models.^14^ The role of ClpP, an additional direct target of ONC201, in DMGs is an area of active investigation.^26^

In addition to single-agent ONC201 responses in adults with recurrent H3 K27M-mutant thalamic glioma,^27^ the first pediatric patient to receive ONC201 following radiation derived a radiographic response, near complete resolution of her Grade IV facial palsy, and hearing restoration.^28^ This patient received ONC201 monotherapy for approximately 12 months, before exhibiting progressive disease outside of the field of prior radiation. The patient received additional radiotherapy for these new lesions and continued on ONC201 for another 34.7 weeks until further progression. She discontinued ONC201 and expired approximately 1 month later (78.2 + weeks on ONC201).^27^

No DLTs were reported in the pediatric population of this trial. The *C*_max_ of ONC201 was moderately lower in this pediatric population (mean 2.3 µg/mL, SD 1.3) compared to adults treated at 625 mg in the phase I trial (mean *C*_max_ 4.29, standard error of mean [SEM], 1.9); however, the *C*_max_ in both trials was within the therapeutic target range.^29^ The mean drug exposure (AUC_0-inf_) in this trial was 16.5 µgh/mL (SD, 9.4) compared to 34.28 (SEM, 21.17) in the adult phase I trial.^29^ T_1/2_ was comparable between pediatric (mean, 8.37 h; SD 6.19) and the adult (9.8; SEM, 1.9) populations. While the peak ONC201 concentration and exposure was lower in this pediatric population, it is notable that the present analysis included all dose levels, whereas the data from the adult trial included only patients receiving the 625 mg dose; this may have accounted for the differences observed. The study did not evaluate doses higher than 625 mg scaled by body weight, which was also the MAD in adults based on saturation of efficacy and pharmacodynamics in preclinical models at allometrically lower doses.^19,20,29^ Higher doses could be evaluated in future studies of ONC201 and increased dosing frequencies are being explored in clinical trials.

The small sample size, population heterogeneity, initiation of ONC201 between radiation and recurrence in the majority of this cohort, and other elements inherent to the phase I design of the study preclude efficacy conclusions. These results support the continued clinical investigation of ONC201 for DMG/DIPG as a well-tolerated investigational therapy. The safety of ONC201 in children with newly diagnosed DIPG when administered concurrently with radiation, as well as pharmacodynamics and intratumoral exposure in pediatric H3 K27M-mutant glioma patients continue to be investigated in separate arms of this clinical trial.

## Supplementary Material

vdac143_suppl_Supplementary_Material
