# Supplementary material for: Phase I dose escalation and expansion trial of single agent ONC201 in pediatric diffuse midline gliomas following radiotherapy
Source: Neurooncol Adv. 2022 Sep 13;4(1):vdac143. doi: 10.1093/noajnl/vdac143 (PMC9639395; doi:10.1093/noajnl/vdac143)

**Supplemental Tables and Figures**

**Supplemental Table 1: Eligibility criteria.**

| **Inclusion Criteria:**   1. Age: 2-19 years. 2. Patient body weight must be above the minimum necessary for the patient to receive the ONC201 dose indicated for the currently enrolling dose level. The minimum body weight ranges from 10 to 35kg depending on the dose level. 3. Patients with glioma who are positive for the H3 K27M mutation (positive testing in CLIA laboratory) and have completed at least one line of prior therapy. Evidence of progression is not required so that ONC201 may be administered to patients in the maintenance setting or to patients with recurrent disease. No more than two prior episodes of recurrence from radiotherapy and/or chemotherapy are allowed. Use of bevacizumab solely for treatment of radiation necrosis, pseudoprogression, or treatment effect will not be considered a recurrence. Post-mortem biopsy is required if H3 K27M status of tumor is unknown and archival tumor tissue not available. 4. Karnofsky performance score ≥50 for patients ≥16 years of age, and Lansky performance score ≥50 for patients <16 years of age. 5. From the projected start of scheduled study treatment, the following time periods must have elapsed: 5 half-lives from any investigational agent, 4 weeks from cytotoxic therapy (except 23 days for temozolomide and 6 weeks from nitrosoureas), 6 weeks from antibodies, or 4 weeks (or 5 half-lives, whichever is shorter) from other anti-tumor therapies. 6. Adequate organ (bone marrow, kidney and liver) function. 7. Ability to understand a written informed consent document, and the willingness to sign it. Assent will be obtained when appropriate based on the patient’s age. 8. All adverse events Grade >1 related to prior therapies (chemotherapy, radiotherapy, and/or surgery) must be resolved to grade 1 or baseline, except for alopecia and sensory neuropathy Grade ≤2, or other Grade ≤2 not constituting a safety risk based on investigator’s judgment, are acceptable. 9. For patients post pubertal: Female patients must agree to use effective contraception during the period of the trial and for at least 90 days after completion of treatment. Male patients must be surgically sterile or must agree to use effective contraception during the period of the trial and for at least 90 days after completion of treatment. The decision of effective contraception will be based on the judgment of the principal investigator. 10. Corticosteroid dose must be stable or decreasing for at least 3 days prior to the baseline MRI scan. 11. Ability to be able to swallow and retain orally administered medication. 12. Archival tumor specimen: All patients in Arm A must submit at least 5 unstained slides from a tumor specimen that harbors H3 K27M mutation. |
| --- |
| **Exclusion Criteria:**   1. Current or planned participation in a study of another investigational agent or using an investigational device. 2. History of allergic reactions attributed to compounds of similar chemical or biologic composition to ONC201 or its excipients. 3. Uncontrolled intercurrent illness including, but not limited to, ongoing or active infection or psychiatric illness/social situations that would limit compliance with study requirements. 4. Any known clinically significant active infection including bacterial, fungal or viral including hepatitis B, hepatitis C or any underlying disease or in the recent past which could compromise enrollment and safety of the patient 5. Known history of cardiac arrhythmias including atrial fibrillation, tachyarrhythmias or bradycardia, unless arrhythmia is controlled and after Cardiology has cleared patient to receive ONC201. Receiving therapeutic agents known to prolong QT interval will be excluded. Patients with a history of congestive heart failure, myocardial infarction, or stroke in the last 3 months will be excluded. 6. Active illicit drug use or diagnosis of alcoholism. 7. Known additional malignancy that is progressing or requires active treatment within 3 years of start of study drug. 8. Concomitant use of potent CYP3A4/5 inhibitors during the treatment phase of the study and within 72 hours prior to starting study drug administration. 9. Concomitant use of potent CYP3A4/5 inducers, which include enzyme inducing antiepileptic drugs, during the treatment phase of the study and within 2 weeks prior to starting treatment. Concurrent dexamethasone is allowed. |

**Supplemental Table 2: ONC201 dose assignment by dose escalation cohort and body weight.** The target adult dose of 625 mg was allometrically scaled as described and rounded to 125 mg intervals (strength of one capsule).

| Patient body weight (kg) | ONC201 (mg) equivalent of 625 mg adult dose^a^ | **ONC201 Dose Level** | | |
| --- | --- | --- | --- | --- |
|  |  | **2** | **1** | **-1** |
|  |  | ONC 201 (mg) to be administered, rounded to capsule strength | | |
| 10 | 145 | 125 | NE | NE |
| 15 | 197 | 250 | 125 | NE |
| 20 | 250 | 250 | 125 | NE |
| 25 | 289 | 250 | 125 | NE |
| 30 | 331 | 375 | 250 | 125 |
| 35 | 372 | 375 | 250 | 125 |
| 40 | 410 | 375 | 250 | 125 |
| 45 | 450 | 500 | 375 | 250 |
| 50 | 486 | 500 | 375 | 250 |
| >50 | -- | 625 | 500 | 375 |

^a^Dose for each weight was calculated using a power model assuming an average adult weight of 70kg and an exponent of ¾ (scaled dose = dose_70kg_(patient weight/70kg)^¾^).^1^

NE, not eligible due to body weight and capsule size.

**References**:

1. Barbour AM, Fossler MJ, Barrett J. Practical considerations for dose selection in pediatric patients to ensure target exposure requirements. *AAPS J*. 2014;16(4):749-55.

**Supplemental Table 3: Clinical outcomes of ONC201-treated pediatric H3 K27M-mutant glioma patients.**

| **Patient population** | **Number of patients** | **PFS**  **Median (range)** | **OS**  **Median (range)** |
| --- | --- | --- | --- |
| Post-radiation, not recurrent | 16 | 20.4 weeks (7.8-129) | 53.8 weeks (25.6-145.5) from diagnosis |
| Recurrent | 6 | 12.6 weeks (4.8-21.3) | 19.6 weeks (4.8-39.5) |

**Supplemental Figure 1:**

Pharmacokinetics of weekly, oral ONC201 in pediatric H3 K27M-mutant glioma patients.

ONC201 plasma concentrations following the first dose of ONC201. Concentrations are shown as the mean for each dose cohort in dose escalation (125 mg, 250 mg, 375 mg, 500 mg and 625 mg). Data points below the limit of detection (1 ng/mL) are excluded from the plots. Each error bar indicates standard deviation.


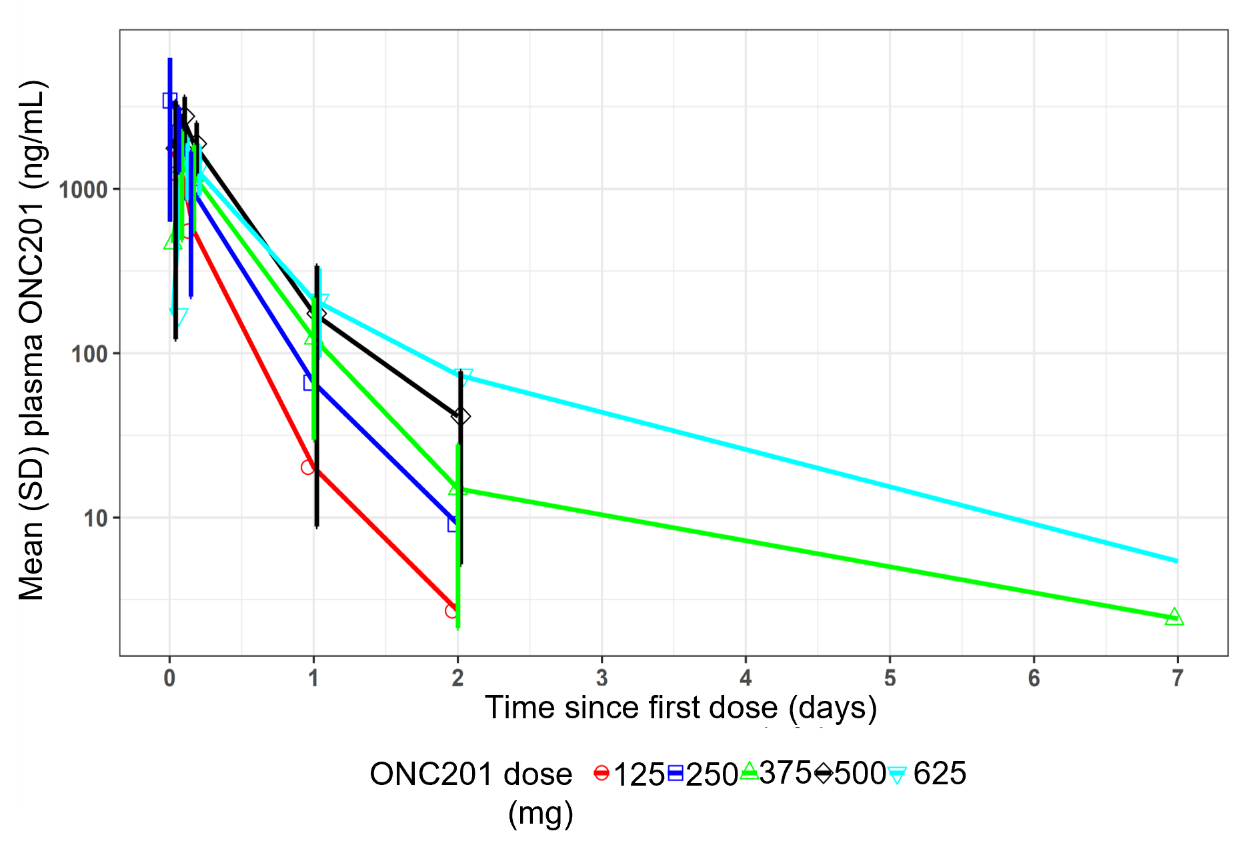


**Supplemental Figure 2:** ONC201 exposure covariance with C_max_. Maximum ONC201 plasma concentrations following the first dose of ONC201. Each dot represents concentration for each patient.


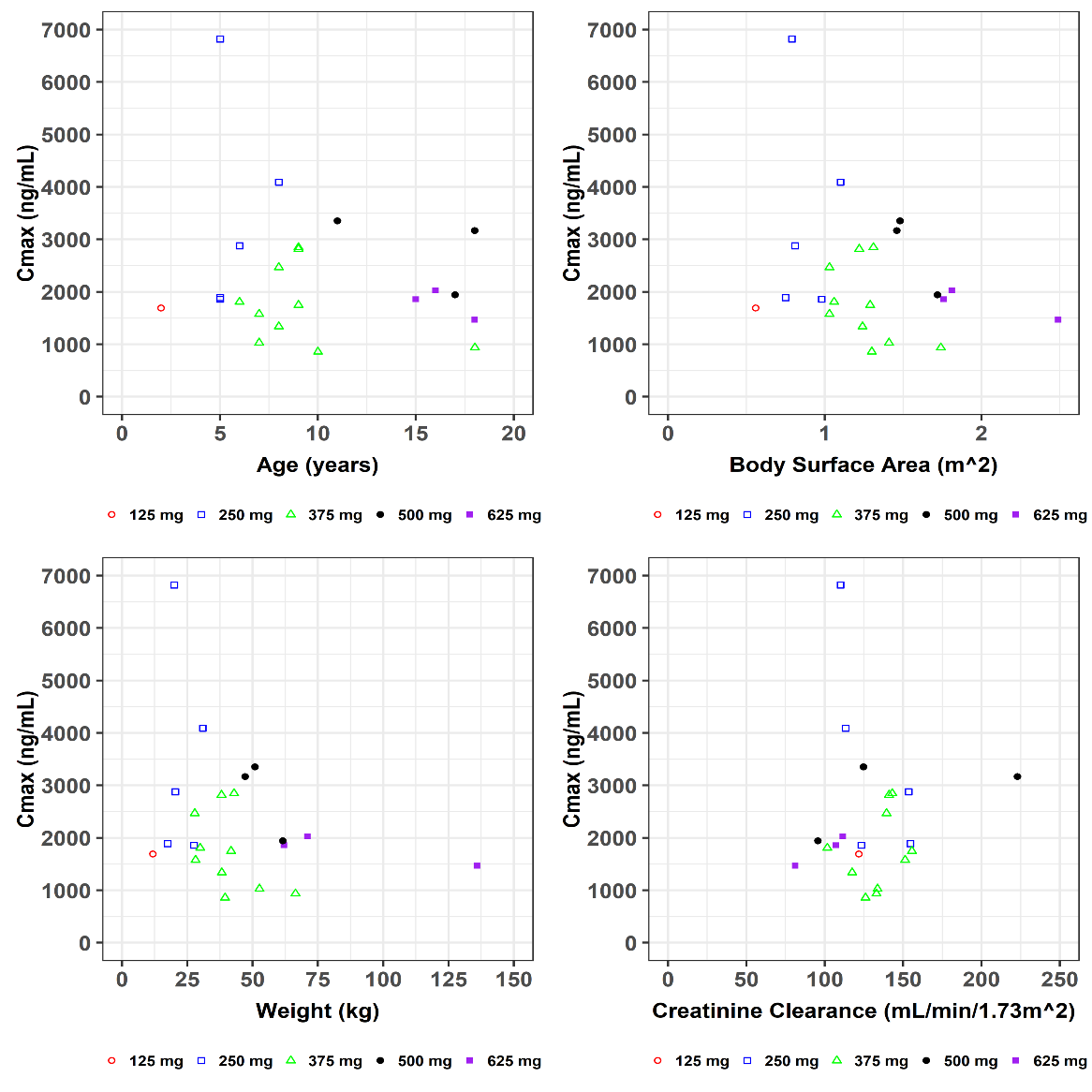

Supplement: vdac143_suppl_Supplementary_Material [file vdac143_suppl_supplementary_material.docx]
